# Respirasome Proteins Are Regulated by Sex-Hormone Interactions in the Brain

**DOI:** 10.3390/ijms232314754

**Published:** 2022-11-25

**Authors:** Andrew J. McGovern, Maria Angeles Arevalo, Sergio Ciordia, Luis Miguel Garcia-Segura, George E. Barreto

**Affiliations:** 1 Department of Biological Sciences, Faculty of Science and Engineering, University of Limerick, V94 T9PX Limerick, Ireland; 2 Instituto Cajal, CSIC, 28002 Madrid, Spain; 3 CIBERFES, Instituto de Salud Carlos III, 28029 Madrid, Spain; 4 Unidad de Proteómica, Centro Nacional de Biotecnología (CNB-CSIC), Cantoblanco, 28049 Madrid, Spain

**Keywords:** sex differences, Tibolone, proteome, mitochondria, respirasome, gonadectomy

## Abstract

The existence of sex differences in disease incidence is attributed, in part, to sex differences in metabolism. Uncovering the precise mechanism driving these differences is an extraordinarily complex process influenced by genetics, endogenous hormones, sex-specific lifetime events, individual differences and external environmental/social factors. In fact, such differences may be subtle, but across a life span, increase susceptibility to a pathology. Whilst research persists in the hope of discovering an elegant biological mechanism to underpin sex differences in disease, here, we show, for the first time, that such a mechanism may be subtle in nature but influenced by multiple sex-specific factors. A proteomic dataset was generated from a gonadectomized mouse model treated with Tibolone, a menopausal hormone therapy. Following functional enrichment analysis, we identified that Alzheimer’s disease and the electron transport chain-associated pathways were regulated by sex-hormone interactions. Specifically, we identified that the expression of three respirasome proteins, NDUFA2, NDUFA7 and UQCR10, is significantly altered by compounding factors that contribute to sex differences. These proteins function in bioenergetics and produce reactive oxygen species, which are each dysregulated in many diseases with sex differences in incidence. We show sex-specific reprogrammed responses to Tibolone following gonadectomy, which primarily influence the expression of proteins contributing to metabolic pathways. This further infers that metabolic differences may underpin the observed sex differences in disease, but also that hormone therapy research now has potential in exploring sex-specific interventions to produce an effective method of prevention or treatment.

## 1. Introduction

Criteria for clinical trials did not include females as necessary subjects until the 1993 [1] and as a result of this, sex difference research is seriously underdeveloped. The complexity of this field is significant, with contributing factors often compounding and potentially interacting. These factors include genetics, sex-specific hormones and temporal events (pubertal timing, age-related testosterone decline, menopause and pregnancy), as well as the influence of our environment/social experience. The biological effects are modelled in male and female animals and cells, which can be treated with or deprived of hormones to explore their impact on cellular mechanisms. The observed impacts may explain sex differences in disease incidence.

Hormonal deprivation, akin to menopause and late-life testosterone decline, is a risk factor for many disorders. For example, Alzheimer’s disease affects twice as many women as men, and menopause is a known risk factor [2] linked to the dysregulation of inflammation and metabolism [3]. Interestingly, menopause also increases the risk of Parkinson’s disease in women [4], even though there are 50% more males with Parkinson’s than females. Given the neurodegenerative nature of these diseases, it is believed that this relationship may be associated with the neuroprotective effects of oestrogens [5,6,7]. However, little is known about reprogrammed responses to hormone replacement therapies following hormonal deprivation, and if such reprogramming is preventable or rescuable. The observed interactions may explain sex differences in disease incidence and give insight into future interventions.

A potential mediator of these sex differences, and the relationship with hormonal deprivation, is the regulation of mitochondrial function by sex hormones, namely oestrogen, progesterone and androgens. This is evident in Alzheimer’s disease and Parkinson’s disease (see review by Ventura-Clapier et al. [8]), which are each associated with differently dysregulated and dysfunctional mitochondria in men and women. Mitochondrial dysfunction is a logical consequence in most cellular pathologies; by the same reasoning, perturbed mitochondrial function may exacerbate the risk of, or incite, cellular pathogenesis, which may explain some sex differences in disease incidence.

Of the many mitochondrial functions we observe as dysregulated, the electron transport chain is in focus here. It constitutes a series of protein complexes which may form sub-complexes or a supercomplex called the respirasome on the inner mitochondrial membrane. Together, the respirasome takes the products of glycolysis and of the Krebs cycle, passing electrons along each protein complex, driving protons out of the mitochondrial matrix and creating the mitochondrial membrane potential. This is essential to the production of ATP in this pathway. This process is critical to cellular productivity and homeostasis as the key producer of ATP, and its management of the damaging waste it produces, such as the production of reactive oxygen species by complex 1 and 3, is of interest. Its dysregulation can lead to an imbalance in energy production and increased cytotoxic conditions, potentially creating conditions more susceptible to pathologies [9].

The dysregulation of mitochondrial function following hormonal deprivation is associated with increased risk of neurodegenerative diseases [10,11]. However, mitochondrial dysfunction can be attenuated when treated with hormone replacement therapies [12], such as Tibolone [13,14,15,16,17]. Tibolone is an FDA-approved hormone replacement therapeutic drug already used to treat symptoms of menopause as a selective progesterone, oestrogen and androgen regulator. The mechanisms of how mitochondrial protection is mediated by Tibolone are not fully explored [5,15], nor is the question of whether it is shared in males and females. In addition, it is not known if the response to Tibolone is consistent or reprogrammed following hormonal deprivation in either sex.

To explore these interactions, a male and female gonadectomised (gonadal hormone deprivation) mouse model was treated with Tibolone. This model can differentiate sex differences which are regulated by adult hormonal influence or genetics and/or postnatal hormonal organisation [18]. Whole-brain proteomic profiling was conducted using prefrontal cortex tissue, and we performed statistical and bioinformatic analyses to detect enriched functions which are impacted by sex, gonads and Tibolone, as well as all interactions.

## 2. Results

To study the impact of key factors in sex differences (genetic/early life organisation and sex hormones) a proteomic dataset was generated from the prefrontal cortex in a gonadectomised model of male and female mice treated with Tibolone. Three samples from each test group underwent protein extraction followed by liquid chromatography with triple-TOF mass spectroscopy. This was then analysed using MaxQuant (v1.10.43) utilizing the integrated Andromeda search engine against the murine Uniprot database. This resulted in 2817 proteins with at least one identified peptide. This dataset was filtered to include two or more valid values per test group, resulting in 1135 proteins. These data were then transformed and missing values were imputed using Perseus. Following this, we performed a three-way ANOVA to identify proteins with significant changes in concentration following the impact of sex (herein defined as mice born male (XY) and female (XX), each with their sex-specific early-life hormonal programming and puberty), gonadectomy (male and female mice that were gonadectomised at 2 months), Tibolone (mice that received a Tibolone injection 24 h before sacrifice), and interactions between each factor (Supplementary File S1). The proteins with *p* < 0.15 and a protein–protein interaction analysis (PPI) followed by functional enrichment were assessed to identify those being regulated by all variables and significant associated clusters.

### 2.1. Sex Regulates Metabolism, Transcription and Organelle Localisation

**Figure 1:**
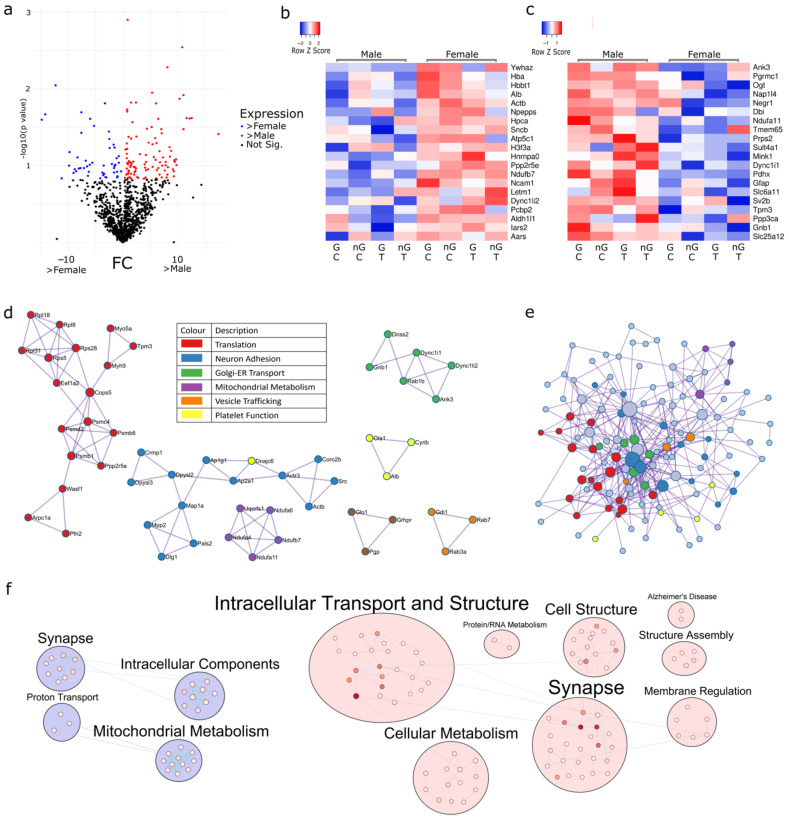
Proteins significantly regulated according to sex. Proteins with sexually dimorphic expression are presented using a volcano plot (**a**); the top 20 upregulated in females (**b**) and males (**c**) are shown in heatmaps. MCODE-driven PPI analysis (**d**) found multiple pathways to be different between males and females, including oxidative phosphorylation (purple, *p* = 1 × 10^−11^), Parkinson’s disease (purple, *p* = 1.58 × 10^−11^), non-alcoholic fatty liver disease (purple, *p* = 2 × 10^−11^), axon guidance (blue, *p* = 1.26 × 10^−10^), nervous system development (blue, *p* = 1.58 × 10^−10^) and metabolism of RNA (red, *p* = 2.5 × 10^−10^). PPI full network enrichment analysis (**e**) identified organelle localisation (*p* = 5 × 10^−11^), establishment of organelle localization (*p* = 1.25 × 10^−9^) and supramolecular fibre organization (*p* = 1.26 × 10^−9^). Functionally enriched terms were clustered and mapped into curated bubbles (**f**). In blue, we see the functions which are upregulated in females, and in pink, those upregulated in males. These functions are noted as potentially sexually dimorphic. Darker nodes indicate lower p values. FC: fold chance, G: gonads, nG: no gonads, C: control, T: Tibolone.

The three-way ANOVA revealed 148 proteins (Figure 1a) as being differentially expressed (*p* < 0.15) in males (Figure 1b) and females (Figure 1c), irrespective of the presence of gonads or Tibolone treatment. There were a total of 49 proteins upregulated in females and 99 proteins upregulated in males. These proteins were then analysed via PPI to identify clusters of functionally enriched pathways impacted by sex. Enriched clusters included transcription and translation (log_10_*p* > −9.6), neuron adhesion (log_10_*p* > −9.9), Golgi-endoplasmic reticulum transport (log_10_*p* > −5.8), mitochondrial metabolism (log_10_*p* > −11), vesicle trafficking (log_10_*p* > −7.1) and platelet function (log_10_*p* > −6.8) (Figure 1d and Supplementary Table S1). When the PPI of the 148 proteins were analysed as a fully connected network organelle localisation (log_10_*p* = −10.3), the establishment of organelle localisation (log_10_*p* = −8.9) and supramolecular fibre organisation (log_10_*p* = −8.7) was significantly enriched (Figure 1e and Supplementary Table S1). Utilising the EnrichmentMap pipeline in Cystoscape, we mapped functional clusters, which showed the functionally enriched landscape of the sex differences (Figure 1f and Supplementary Table S2).

### 2.2. Gonadectomy Impacts Cellular Functions, including Endocytosis, Translation and Carbon Metabolism

**Figure 2:**
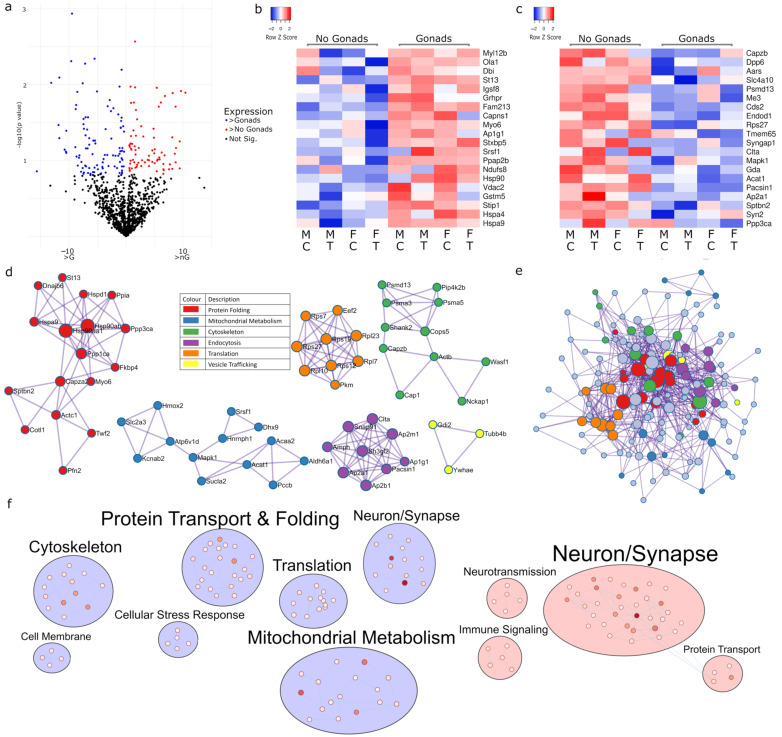
Regulation of proteins in the presence or absence of gonads. The impact of gonads on expression is presented using a volcano plot (**a**), with the top twenty upregulated proteins in the presence of gonads (**b**) and in their absence (**c**). PPI analysis with MCODE (**d**) identified several enriched functions, including receptor- and clathrin-mediated endocytosis (purple, *p* = 6.31 × 10^−15^), ribosomes (orange, *p* = 1 × 10^−13^), protein folding (red, *p* = 3.16 × 10^−13^), propanoate metabolism (blue, *p* = 3.16 × 10^−9^), UCH proteinases (green, *p* = 1.26 × 10^−7^) and membrane trafficking (yellow, *p* = 1.6 × 10^−5^). Full network analysis (**e**) revealed enriched functions in the regulation of protein polymerisation (*p* = 5 × 10^−11^), the regulation of protein-containing complex assembly (*p* = 6.31 × 10^−11^) and carbon metabolism (*p* = 1.6 × 10^−10^). Proteins with increased expression in the presence or absence of gonads were analysed for functional enrichment, and then, mapped together based on shared function (**f**). Darker nodes indicate lower *p* values. FC: fold chance, M: male, F: female, C: control, T: Tibolone.

Examining the impact of gonads, or a lack thereof, is a critical exploration in sex difference research as the functional role of gonads has temporal and developmental impacts on sex differences. Here, we found 174 proteins (Figure 2a) with differential expression (*p* < 0.15) in mice with gonads (Figure 2b) and without (Figure 2c), and we analysed which pathways were impacted across both males and females by gonadectomy irrespective of Tibolone or sham treatment. PPI analysis revealed enriched clusters in protein folding (log_10_*p* > −12.5), mitochondrial metabolism (log_10_*p* > −8.5), the cytoskeleton (log_10_*p* > −6.9), endocytosis (log_10_*p* > −14.2), translation (log_10_*p* > −13) and vesicle trafficking (log_10_*p* > −4.8) (Figure 2d and Supplementary Table S3). PPI analysis of a fully connected cluster found the regulation of protein polymerization (log_10_*p* = −10.3), protein-containing complex assembly (log_10_*p* = −10.2) and carbon metabolism (log_10_*p* = −9.8) to be significant (Figure 2e). Mapping the enriched functions into clusters allows us to see that changes in gonadectomized mouse protein expression appear to be associated with altered protein transport and folding, translation and mitochondrial metabolism, whilst gonads appear to influence neurotransmission and immune signalling (Figure 2f, Supplementary Table S4).

### 2.3. Multiple Pathways in Mitochondrial Metabolism Can Be Targeted with Tibolone

**Figure 3:**
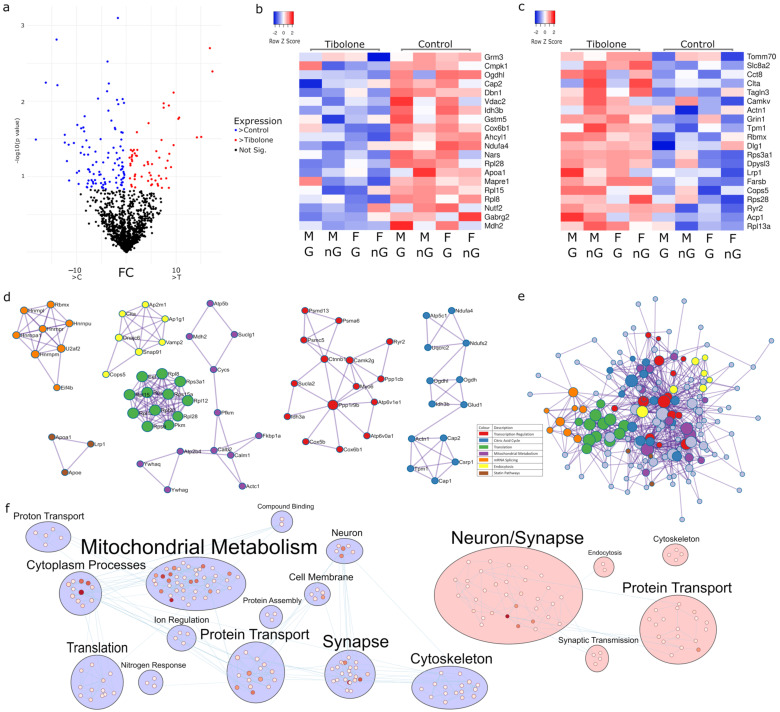
Hormone-regulated proteins in the prefrontal cortex of mice. A single Tibolone treatment produced the greatest number of differentially expressed proteins (**a**). The top 20 proteins expressed in the control (**b**) and in Tibolone (**c**) are shown in heatmaps. The first MCODE-driven PPI analysis (**d**) revealed SRP-dependent cotranslational protein targeting to the membrane (green, *p* = 7.94 × 10^−23^), Nonsense-Mediated Decay (NMD) independent of the Exon Junction Complex (EJC) (green, *p* = 1 × 10^−22^), the formation of a pool of free 40S subunits (green, *p* = 2.5 × 10^−22^), mRNA splicing (orange, *p* = 2 × 10^−14^), the citric acid (TCA) cycle and respiratory electron transport (blue, *p* = 5 × 10^−10^) and clathrin-mediated endocytosis (yellow, *p* = 2.5 × 10^−10^) to be the most impacted functions upon Tibolone treatment. When PPI analysis was performed on all proteins in one network (**e**), generation of precursor metabolites and energy (*p* = 1.26 × 10^−12^) and Alzheimer’s disease (*p* = 5.01 × 10^−12^) were the most enriched functions. When all of the significantly enriched functions were mapped into clusters (**f**), we can see a large number of mitochondrial metabolism functions being more prevalent in the control group (blue), whilst neuronal and synapse-associated functions are more upregulated upon Tibolone treatment.

Tibolone is a menopausal hormone therapy that is a selective progesterone, oestrogen and androgen regulator. Our analysis found 167 proteins regulated by Tibolone in male and female mice, with and without gonads (Figure 3a), with *p* < 0.15. Of these, 106 proteins were upregulated (Figure 3b) and 61 proteins were downregulated (Figure 3c). PPI analysis revealed transcription (log_10_*p* > −6.8), the citric acid cycle (log_10_*p* > −9.3), translation (log_10_*p* > −22.1), mitochondrial metabolism (log_10_*p* > −6.3), mRNA splicing (log_10_*p* > −13.7), endocytosis (log_10_*p* > −9.6) and statin pathways (log_10_*p* > −9.2) as being significant (Figure 3d and Supplementary Table S5). Analysis of these proteins in a fully connected PPI network found enrichment in the generation of precursor metabolites and energy (log_10_*p* = −11.9) and Alzheimer’s disease (log_10_*p* = −11.3) (Figure 3e). Enrichment mapping showed that mitochondrial metabolism and protein transport appear to be central targets following Tibolone’s impact on protein expression (Figure 3f, Supplementary Table S6).

### 2.4. Two-Way Interactions of Sex-Associated Factors

#### 2.4.1. Sex and Gonadectomy Interact to Regulate Nonsense-Mediated Decay and Metabolism

**Figure 4:**
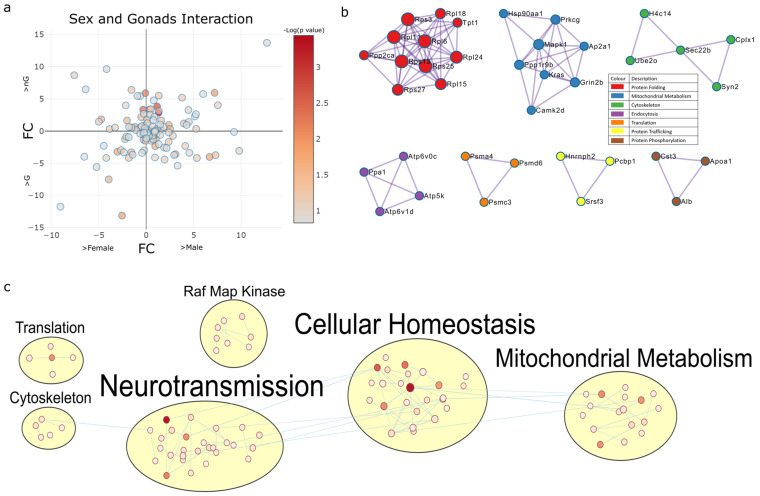
Two-way interaction of proteins regulated by sex–gonad interactions. (**a**) A total of 119 proteins were identified to be regulated by the interaction between sex and gonads. (**b**) PPI analysis of these proteins found Nonsense-Mediated Decay (NMD) enhanced by the Exon Junction Complex (EJC) (red, *p* = 1.56 × 10^−22^), respiratory electron transport (blue, 3.16 × 10^−17^), cytoplasmic ribosomal proteins (green, *p* = 6.3 × 10^−14^), the proteosome (orange, *p* = 1 × 10^−8^), mRNA splicing (yellow, *p* = 6.3 × 10^−7^) and post-translational protein phosphorylation (brown, *p* = 1.58 × 10^−7^) to be the top enriched functions, per cluster. PPI analysis without MCODE-driven clustering revealed Nonsense-Mediated Decay to be the most enriched function (*p* = 3.16 × 10^−10^) regulated by sex and gonads. (**c**) Functions enriched by sex–gonad interactions were also grouped by observing functions associated with RAF-MAP kinase. Of these enriched functions, the most significant GO cellular component was the cytoplasm (*q* = 4.14 × 10^−17^), the most significant GO molecular function was identical protein binding (*q* = 2.92 × 10^−7^), the most significant reactome pathway was metabolism (*q* = 1.66 × 10^−5^), the most significant KEGG pathway was endocrine and other factor-regulated calcium reabsorption (*q* = 2.63 × 10^−5^) and the most significant GO biological process was central nervous system development (*q* = 1 × 10^−4^).

Next, we analysed proteins whose expression was identified to be regulated based on the interaction between sex, gonads, and/or Tibolone (*p* < 0.15). A total of 119 proteins were identified to have their expression impacted by the interaction of sex and gonads, irrespective of Tibolone status (Figure 4a). PPI analysis found energy integration (log_10_*p* > −8.4), the electron transport chain (log_10_*p* > −16.5), ribosomes (log_10_*p* > −13.2), transcription (log_10_*p* > −12.2), prion diseases (log_10_*p* > −7.8), translation (log_10_*p* > −8), long-term potentiation (log_10_*p* > −10.8), oxidative phosphorylation (log_10_*p* > −8.8), proteosomes (log_10_*p* > −8), mRNA splicing (log_10_*p* > −6.2) and post-translation modifications (log_10_*p* > −6.8) to be significant (Figure 4b and Supplementary Table S7). The enrichment map clustering shows neurotransmission, cellular homeostasis and mitochondrial metabolism-associated pathways as being enriched by sex–gonad interactions (Figure 4c and Supplementary Table S8).

#### 2.4.2. Sex–Tibolone Interactions Impact Complex 1 of the Electron Transport Chain

**Figure 5:**
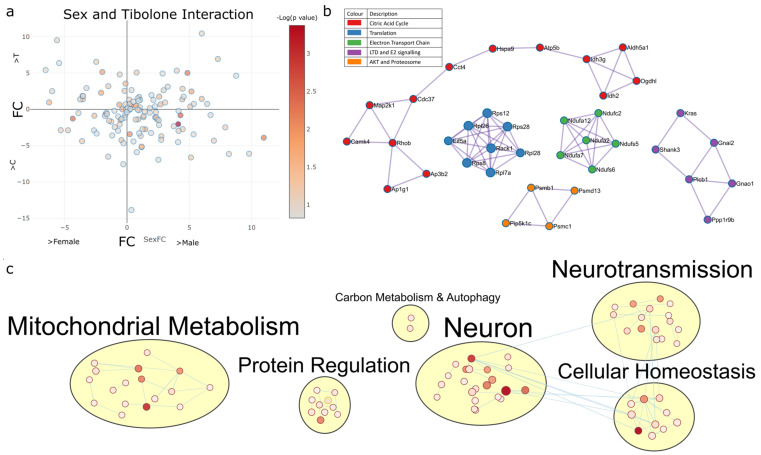
Functional enrichment predicts complex 1 protein regulation to have sex-specific responses to Tibolone. (**a**) A total of 132 proteins were found to be regulated by sex–Tibolone interactions. (**b**) PPI analysis of proteins regulated by sex–Tibolone interactions identified respiratory chain complex I, mitochondria (green, *p* = 2.5 × 10^−17^), cytoplasmic ribosomal proteins (blue, *p* = 2.5 × 10^−13^), long-term depression (purple, *p* = 1 × 10^−9^), PIP3 activation of AKT signalling (orange, *p* = 1.26 × 10^−8^) and the citrate cycle (TCA, krebs cycle) (red, *p*= 3.98 × 10^−7^) to be the most enriched functions in each cluster. Fully connected PPI analysis found Parkinson’s disease (*p* = 3.98 × 10^−10^) to be the most significantly enriched function regulated by sex–Tibolone interactions. (**c**) When enriched functions were mapped, carbon metabolism- and autophagy-associated functions were identified to be uniquely regulated by sex–Tibolone interactions. The GO cellular component cytoplasm (*q* = 1.22 × 10^−23^), KEGG pathway Alzheimer’s disease (*q* = 5.17 × 10^−10^), reactome pathway metabolism (q = 1.23 × 10^−8^), GO molecular function identical protein binding (*q* = 1.01 × 10^−7^) and GO biological process regulation of biological quality (*q* = 5.23 × 10^−7^) were the functions most enriched by sex–Tibolone interactions in each enrichment category.

A total of 132 proteins were found to have their expression impacted by the interaction of sex and Tibolone treatment in mice with and without gonads (*p* < 0.15) (Figure 5a). PPI analysis identified pathway clusters, including the citric acid cycle (log_10_*p* > −6.4), translation (log_10_*p* > −12.6), respiratory complex 1 (log_10_*p* > −16.6), long-term depression (log_10_*p* > −9) and proteosomes (log_10_*p* > −7.9) (Figure 5b and Supplementary Table S9), with the enrichment map clusters showing mitochondrial metabolism, protein regulation and neuronal function and cell homeostasis (Figure 5c and Supplementary Table S10).

#### 2.4.3. Gonadectomy and Tibolone Interact to Regulate Translation and Post-Translation-Associated Pathways

**Figure 6:**
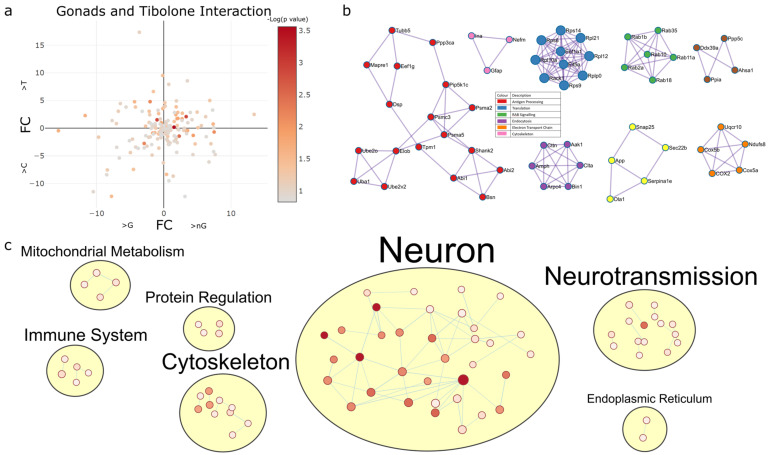
Gonadectomy alters protein expression related to RAB geranylgeranylation, ribosomes and ATP synthesis following Tibolone treatment. (**a**) A total of 163 proteins were found to be regulated by gonad–Tibolone interactions. (**b**) PPI analysis of proteins regulated by gonad–Tibolone interactions found that RAB geranylgeranylation (green, *p* = 3.16 × 10^−16^), ribosomes (blue, *p* = 3.16 × 10^−13^), ATP synthesis-coupled electron transport (orange, *p* = 3.16 × 10^−13^), clathrin-mediated endocytosis (purple, *p* = 6.3 × 10^−11^), antigen processing, ubiquitination and proteasome degradation (red, *p* = 2.5 × 10^−9^) and intermediate filament cytoskeleton organization (pink, p = 1.58x10^−8^) were the most enriched functions in each cluster. When PPI analysis was performed without clustering, the regulation of protein polymerisation was most significantly enriched (*p* = 1.25 × 10^−8^). (**c**) When enriched functions regulated by gonad–Tibolone interactions were mapped into groups, we noted that more functions associated with the immune system and endoplasmic reticulum were evident than in sex–gonad or sex–Tibolone functional enrichment analysis. The most enriched GO cellular component was the synapse (*q* = 1.49 × 10^−27^), the most enriched GO molecular function was structural molecule activity (*q* = 9.36 × 10^−11^), the most enriched GO biological process was regulation of cellular component organisation (*q* = 6 × 10^−10^), the most enriched reactome pathway was the immune system (4.95 × 10^−5^) and the most enriched KEGG pathway was Alzheimer’s disease (*q* = 0.0011) in response to gonad–Tibolone interactions.

The analysis of gonad–Tibolone interactions found 165 proteins with *p* < 0.15 to have gonad dependent responses to Tibolone in male and female mice (Figure 6a). PPI analysis of these proteins revealed enriched pathways involved in antigen processing (log_10_*p* > −8.6), translation (log_10_*p* > −12.5), RAB signalling (log_10_*p* > −15.5), endocytosis (log_10_*p* > −10.2), the electron transport chain (log_10_*p* > −12.5) and the cytoskeleton (log_10_*p* > −7.8) (Figure 6b and Supplementary Table S11), and the enrichment map shows notable clusters of enrichment items in neuronal processes, neurotransmission, the cytoskeleton and the immune system (Figure 6c and Supplementary Table S12).

### 2.5. Three-Way Interaction of Sex, Gonads and Tibolone Implicate Connection to Respiration and Alzheimer’s Disease

**Figure 7:**
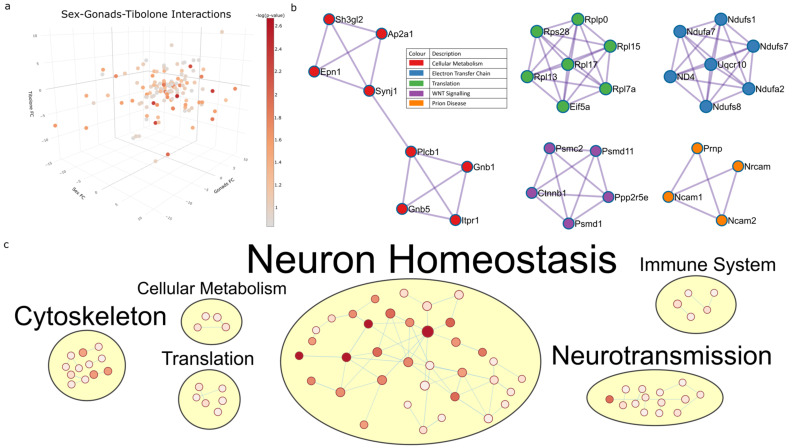
Three-way interaction of proteins regulated by sex, gonads and Tibolone. A total of 139 proteins were identified to be regulated by sex–gonad–Tibolone interactions (**a**). PPI analysis of these proteins (**b**) identified respiratory electron transport (blue, *p* = 3.16 × 10^−17^) to be the most significant function and MCODE node cluster. This was followed by cytoplasmic ribosomal proteins (green, *p* = 6.3 × 10^−14^), the degradation of beta-catenin by the destruction complex (purple, *p* = 6.3 × 10^−13^), the regulation of insulin (red, *p* = 3.98 × 10^−9^) and prion diseases (orange, *p* = 1.58 × 10^−8^). When PPI analysis was performed on all proteins as a single cluster, Alzheimer’s disease was the most significant function (*p* = 2.5 × 10^−10^). Enriched functions were mapped into related groups (**c**) in which the most significant functions regulated by sex–gonad–Tibolone interactions per category were the synapse (*q* = 1.52 × 10^−34^) in GO cellular components, transport (*q* = 4.68 × 10^−8^) in the GO biological process, Alzheimer’s disease and retrograde endocannabinoid signalling (*q* = 5.77 × 10^−8^) in KEGG pathways, metabolism (*q* = 2.18 × 10^−5^) in reactome pathways and structural molecule activity (*q* = 7.51 × 10^−5^) in GO molecular function.

The final result of the three-way ANOVA is proteins whose expression is affected by the interaction between sex, gonads and Tibolone (Supplementary Figure S1). A total of 140 proteins were identified to have their response to Tibolone regulated in a sex- and gonad-specific manner (*p* < 0.15) (Figure 7a, Supplementary Figure S1). PPI analysis identified enriched pathways involved in cellular metabolism (log_10_p > −8.4), the electron transport chain (log_10_*p* > −16.5), translation (log_10_*p* > −13.2), WNT signalling (log_10_*p* > −12.2) and prion disease (log_10_*p* > −7.8) (Figure 7b and Supplementary Table S13). The enrichment map shows a large cluster of enriched pathways involved in neuronal homeostasis (Figure 7c and Supplementary Table S14).

### 2.6. Meta-Analysis for Enriched Functions amongst Proteins Impacted by Sex-Specific Factors Reveals Connection to Alzheimer’s Disease

**Figure 8:**
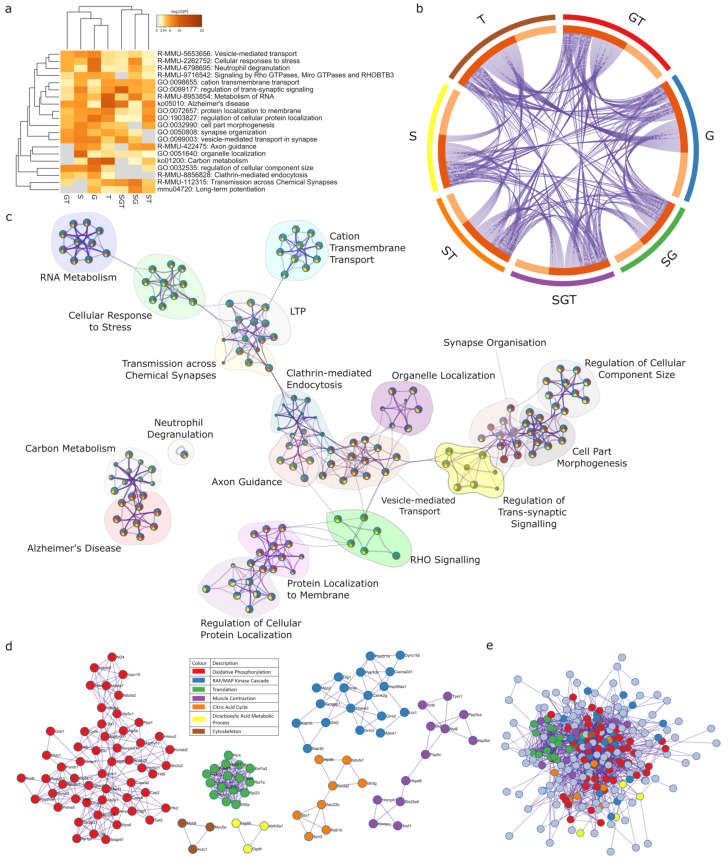
Meta-analysis of proteins significantly regulated by sex, gonads and Tibolone. The proteins identified as being regulated by sex, gonads or Tibolone, as well as all potential interactions, were input into Metascape for functional enrichment analysis, and then, cross referenced to see which functions were significantly enriched across the most protein lists. A heatmap of the top 20 most enriched functions across each list (**a**) is shown, with darker squares indicating higher statistical significance. The 5 most significant functions identified were Alzheimer’s disease (*q* = 4.68 × 10^−23^), metabolism of RNA (*q* = 1.78 × 10^−22^), cellular response to stress (*q* = 9.55 × 10^−20^), regulation of cellular protein localization (*q* = 7.24 × 10^−19^) and vesicle-mediated transport in synapse (*q* = 1.07 × 10^−18^). A Circos plot (**b**) is shown to visualize the number of shared proteins between each protein list and a network of enriched functions (**c**) grouped into clusters represented by the most statistically significant function in that cluster. The pie chart colours within each node correspond to the protein list in which the function is enriched, as per the colours on the Circos plot. All protein lists were then merged and a PPI analysis with MCODE clustering was performed (**d**); it found SRP-dependent cotranslational protein targeting to the membrane to be the most statistically enriched function overall (green, *p* = 1.99 × 10^−23^), followed by oxidative phosphorylation in the red cluster (*p* = 1.99 × 10^−23^), RAF activation in blue (*p* = 1.99 × 10^−8^), dicarboxylic acid metabolic process in yellow (*p* = 7.94 × 10^−8^), then, actin filament-based movement in brown, as well as aerobic respiration in yellow (*p* = 2.5 × 10^−7^) and muscle system process in purple (*p* = 2.5 × 10^−6^). When this analysis was performed without MCODE clustering (**e**), it revealed aerobic respiration (*p* = 5.01 × 10^−14^) and Alzheimer’s disease (*p* = 2.5 × 10^−13^) to be significantly enriched functions.

A meta-analysis of the proteins impacted by sex and hormone effects (gonadectomy and Tibolone), and interactions on protein expression was performed utilizing Metascape, an online pipeline for analysing multiple profiles (Figure 8a). We identified a total of 666 proteins from the seven lists of proteins whose expression is regulated by sex, gonads, and Tibolone, as well as sex–gonad, sex–Tibolone, gonad–Tibolone, and Sex–gonad–Tibolone interactions (Supplementary File S2). In the Circos plot, 174 proteins appear in two protein lists, 64 proteins appear in three lists, 19 proteins appear in four lists and 4 proteins appear in five lists (Figure 8b, Supplementary Table S15, and Supplementary File S3). The top 20 enriched clusters of related pathways and processes were then networked and organised, whereby the most significant component of each cluster was chosen as the name (Figure 8c, Supplementary Table S16, and Supplementary File S4).

The most significantly enriched process and pathway cluster were led by ‘Alzheimer’s disease’, containing mostly enriched functions and processes associated with mitochondrial metabolism, especially the respirasome. ‘Alzheimer’s disease’ is a KEGG pathway with 175 associated proteins. This analysis found that this pathway was enriched in each of the analysed protein lists, with 44 proteins (6.6% of the total protein list analysed in Metascape) from the pathway included across the lists. This pathway was determined to be highly enriched, with log_10_*p* = −26.31, which, following multiple test adjustments was corrected to log_10_*q* = −22.3. The second most enriched function in this cluster, and overall, was ‘the citric acid cycle and respiratory electron transport’ (Supplementary Table S17). This reactome set is associated with 169 proteins in total, 43 of which (6.5% of the protein list) are shared. The significance of this term across the multiple protein sets is log_10_*p* = −25.9495, which was adjusted to log_10_*q* = −22.145 following multiple test corrections.

PPI analysis was then performed on all of the merged lists, which created clusters of proteins contributing to oxidative phosphorylation (log_10_*p* > −13.1), the RAF/MAP kinase cascade (log_10_*p* > −7.2), translation (log_10_*p* > −22.3), muscle contraction (log_10_*p* > −4.1), the citric acid cycle (log_10_*p* > −6.1), the dicarboxylic acid metabolic process (log_10_*p* > −7.1) and the cytoskeleton (log_10_*p* > −4.4) (Figure 8d and Supplementary Table S18). PPI analysis of all the merged lists revealed aerobic respiration (log_10_*p* > −13.3) and Alzheimer’s disease (log_10_*p* > −12.6) to be significantly enriched (Figure 8e and Supplementary Table S18).

### 2.7. NDUFA2, NDUFA7 and UQCR10 Are Regulated by Sex–Gonad–Tibolone Interactions with Relevance to Alzheimer’s Disease

**Figure 9:**
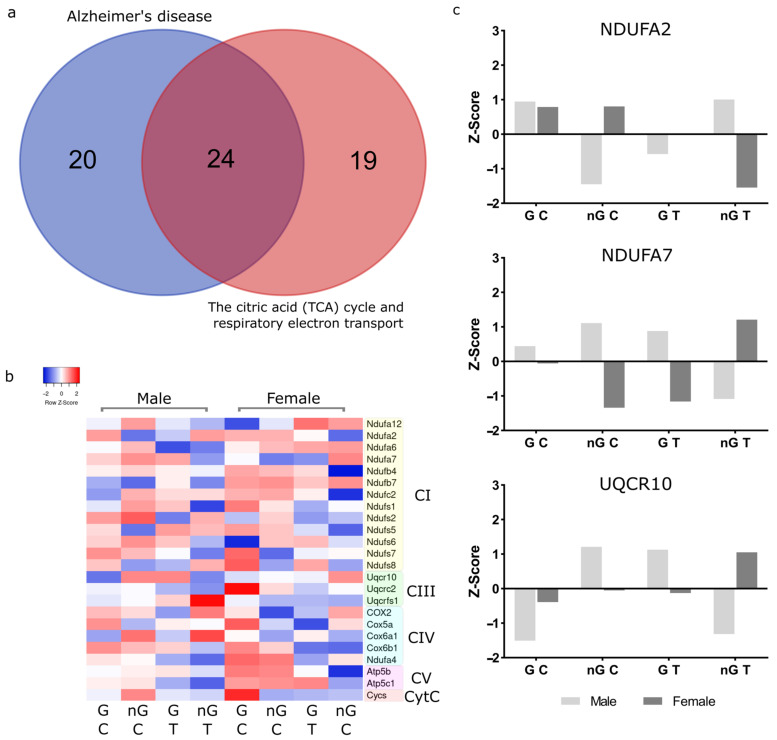
Metascape output and top regulated proteins in relation to sex, gonads and Tibolone. The top two enriched functions from the Metascape analysis of all protein lists were Alzheimer’s disease and the citric acid cycle and respiratory electron transport chain, which both belonged to the same cluster. We identified the 24 proteins shared between these functions (**a**) and members of the respirasome. A heatmap of the 24 respirasome proteins (**b**) was then produced, identifying members of complex 1, 3, 4 and 5 and cytochrome c. Only three of these proteins (**c**) were found to be significantly regulated by sex–gonad–Tibolone interactions: NDUFA2 (*p* = 0.042) and NDUFA7 (*p* = 0.0047) belonging to complex 1, and UQCR10 (*p* = 0.043), which belongs to complex 3 of the respirasome.

In the two most enriched functions, there is an overlap of 24 proteins (Figure 9a), all of which are native to the respirasome (Figure 9b). To explore which of these proteins are most influenced by sex, gonads and Tibolone we crossed proteins in this list that appeared in more than one protein list (sex, gonads, Tibolone or two-way interactions) with proteins that had *p* < 0.05 for sex–gonad–Tibolone interactions. The proteins identified were NDUFA2 (*p* = 0.042), NDUFA7 (*p* = 0.0047) and UQCR10 (*p* = 0.043) (Figure 9c). NDUFA2 and NDUFA7 both belong to complex 1 in the respirasome. Interestingly, NDUFA2 expression is lower in both gonadectomised and Tibolone-treated males, but Tibolone attenuates this altered expression in gonadectomised males. However, gonadectomy does not alter expression in females, but Tibolone exacerbates, then appears to reduce expression. NDUFA7 appears to be upregulated by gonadectomy and Tibolone, but Tibolone may reduce expression in hormonally deprived males. In females, expression is reduced by gonadectomy and Tibolone, but the expression is increased in combination. UQCR10 is a complex 3 protein. Alterations to hormonal activity by gonadectomy and Tibolone treatment increase expression in males, but levels return close to the baseline when Tibolone is given to gonadectomized males. However, we observe small increases in expression following gonadectomy and Tibolone treatment, but in combination, expression is dramatically increased. In summary, the action of Tibolone treatment on respirasome protein expression is sexually dimorphic and dependent on the presence of gonads in adult mice.

## 3. Discussion

Sex differences in neurological health and disease are underpinned, in part, by a number of biological factors which are genetic, hormonal or temporal in affect. The complexity of these compounding factors is substantial; as such, to understand diseases with sex inequality in incidence, further efforts must be made. This pressure is due to a lack of foresight in prior research design, as studies on sex differences in biological science may need to reconsider their design, and certainly adjust their analyses [19,20]. Presently, we continue to lack an understanding of the underpinning mechanisms of sex differences and lack sex-specific interventions as a result. Untangling these biological contributors to sex differences will require meaningful adjustments to the present standards, but the potential benefits of more precise personalized interventions or treatment for disorders in men and women are unquantifiable.

### 3.1. Functional Enrichment Analysis Reveals Altered Pathways Associated with Alzheimer’s Disease and the Electron Transport Chain

To untangle some of these differences, we ran a proteomic analysis of the prefrontal cortex of an adult (male and female) gonadectomized (gonadal hormone deprivation) mouse model with a hormonal supplement (Tibolone). This allows us to interpret if changes in protein concentration between the sexes are regulated by gonadal hormones in adulthood or if such differences are influenced by sex-specific genetics/early-life hormonal organisation, and if there is potential to attenuate unwanted changes with hormonal supplementation [18]. Our analysis aimed to identify patterns of change in protein expression, with respect to function, which are affected by sex, hormonal deprivation and supplementation, as well as how each of these factors may interact. We then utilized Metascape [21] to reveal functionally enriched processes and pathways which appear most often amongst the multiple analyses of proteins impacted by sex, hormonal deprivation and supplementation, and their interactions, to identify the pathways that are most impacted by compounding biological factors contributing to sex differences.

This analysis revealed the most significant cluster of enriched processes to contain many pathways associated with mitochondrial metabolism. The most enriched process overall was ‘Alzheimer’s disease’, which was followed by ‘the citric acid (TCA) cycle and respiratory electron transport’. Specifically, 24 respirasome proteins, whose expression was impacted by sex-associated factors and their interactions, were identified as shared between these two enriched processes. Of these proteins, the expression of three, NDUFA2, NDUFA7 and UQCR10, was identified to belong to the list of proteins significantly (*p* < 0.05) affected by sex–gonad–Tibolone interactions, as well as being present in a shared additional protein list of these top two enriched functions. Together, we believe this bolsters their potential as significant contributors to sex differences and their potential as novel sex-specific regulators of function.

### 3.2. Complex 1 Proteins Are Regulated by Sex-Hormone Interactions

The NDUFA2 and NDUFA7 proteins belong to complex I, the largest protein-complex in the respirasome. This complex is where electrons first enter the electron transport chain and are then transferred from NADH to quinone, which is implicated in the production of reactive oxygen species [22]. This process occurs at the N-module of the complex in which NDUFA2 and NDUFA7 contribute to function.

The precise function of NDUFA2 continues to lack clarity but it has been shown as an essential structural subunit in the N module subcomplex [23], containing a thioredoxin-like fold. Mutations to this subunit are associated with Leigh syndrome which is associated with reduced complex 1 function, assembly and mitochondrial depolarisation [24]. Mutations in NDUFA2 have also been reported to be associated with complex 1 deficiency resulting in leukoencephalopathy [25,26]. Reduced NDUFA2 expression has also been identified as a potential biomarker for Alzheimer’s disease, with an associated immune infiltration [27]. Other studies have shown that deregulated expression of NDUFA2 is associated with mitochondrial dysfunction which can contribute to the development of a head or neck paraganglioma [28]. Interestingly, metformin, a complex 1 inhibitor, corrected this deregulation which was associated with better therapeutic outcomes [28]. Together, we can infer that NDUFA2 is an important subunit to complex 1 metabolic function which makes it again a protein of interest as a potential effector for sex differences in neurodegeneration.

The biological function of NDUFA7 has recently been uncovered in a study that showed that NDUFA7 knockout led to cardiac hypertrophy, indicating that depletion of NDUFA7 resulted in increased production of reactive oxygen species and calcineurin signalling [29]. Notably, cardiac hypertrophy is a disease with worse risk in post-menopausal women which can be attenuated in-part with hormonal supplement [29,30]. Recently, Xiao et al. [31] has shown that mitochondrial micro peptide can bind to NDUFA7 and inhibit complex 1 activity. NDUFA7 has also been associated with the development of rheumatoid arthritis, which was suggested to be mechanistically driven by reactive oxygen species [32]. There are twice as many men as women with rheumatoid arthritis and menopause is a known risk factor for onset, although early menopausal onset was associated with lower severity of the disease indicating that, although sex hormones may directly increase the risk of arthritis, it does not drive the degree of severity [33]. Together this suggests NDUFA7 is an essential protein that regulates complex 1 activity.

### 3.3. Complex 3 Protein UQCR10 Is Regulated by Sex-Hormone Interactions

UQCR10 is a functional protein in complex 3, an important structural subunit that associates with other subunits such as CYC1 to facilitate electron transport and signal transmission. This is dysregulated when the protein is oxidised [34]. This complexome is also an essential step is connecting the functionality of complex 3 and 4 [35]. Early functional exploration indicates it may have unique regulatory functions [36]. Interestingly, recent studies have indicated UQCR10 as playing a role in Alzheimer’s disease pathophysiology [37]. Higher UQCR10 expression is associated with better survival times in lung adenocarcinoma and lung squamous cell carcinoma [38]. Together, although research into the functionality of UQCR10 is still early we are seeing signals that it may be a significant contributor to regulating the functionality of the respirasome. Previous studies have shown that hormonal deprivation, such as in ovariectomy, can disrupt metabolic processes which can be ameliorated by Tibolone [39]. A fascinating metabolomic study by Chabrun et al. showed the greater dysregulation of metabolites following gonadectomising in females than males, as well as the region-specific effects [40].

### 3.4. Future Studies and Limitations

Our study is the first to show that sex, gonadal hormones and Tibolone interact. We also show that gonadectomy produces sex-specific reprogramming which alters the response to Tibolone, and the enrichment meta-analysis implicates cerebral respirasome expression response to Tibolone to be sex and gonadal hormone status dependent. Investigating the validity of sex-hormone interactions regulating the respirasome is critical, as is investigating the impact on metabolic function, as the implied results could enlighten much of our understanding of sex differences in disease and hormonal therapeutics. Mitochondrial dysfunction is continuously associated with many diseases with sex differences in incidence [41,42,43], and the respirasome’s role in energy and waste production makes it a supercomplex of interest. Given the sexual dimorphism between the male and female mitochondrial metabolism of energy [44], which may be managed by oestrogens [45], it is promising to now suggest that these differences are interrupted by menopause (comparable to gonadal hormone deprivation) and dysregulate mitochondria, producing an unbalanced environment, which may be more susceptible to a cellular pathology. If this is the case, hormonal interventions such as Tibolone may be most appropriately investigated as a preventative measure for cellular pathologies, such as Alzheimer’s disease, as it may not directly reverse a pathology, but instead, maintain an environment in which a pathological process is most unlikely to progress.

Fundamentally, what must take precedence is improving how we study drugs and diseases. Hormone replacement therapies have undergone an explosion of growth in prescription since the 1960s for menopause, peaking in the 2000s; this was followed by a drop following panic [46] around early evidence of a poor risk-to-benefit ratio, resulting in a drop in use [47]. Since then, we have seen their use expand further into contraception, hair loss prevention, osteoporosis and coronary disease prevention, as well as applications in transgender medicine, inferring that further characterisation of the admittedly complex sex-specific interactions between people and hormonal drugs is needed. Studies should be conducted which go beyond solely observing genetics and wild-types, but into temporal events such as puberty, pregnancy, menopause or orchiectomy/ovariectomy. Such work could improve experimental design by widely identifying which areas of study even require an observation of sex differences in design, and for those that do, which aspects of sex should be analysed. A recent study showed that only 12.5% of randomized clinical trials on Alzheimer’s disease drugs reported sex-stratified results (although this is improving) [48]. Less than 15% of the studies analysed described methods to even observe sex differences [49]. Given the sex difference in the incidence of Alzheimer’s disease (2:1 females to males), in the context of the contradicting impacts of sex-specific factors on mitochondrial metabolism pathways associated with neurodegenerative pathologies found in this study, we must consider that there may be a therapeutic intervention which is effective for women but not men (and vice versa), which we may still discover or, frighteningly, which we may have missed. More informed experimental evidence and design would increase how informed patients are about their benefits and risks and inform clinical professionals to make more precise therapeutic strategies.

It is important to acknowledge that in this study, we set the alpha value for the inclusion of proteins in the functional enrichment and PPI analysis at 0.15. Our sample size (*n* = 3) may lack the statistical power to detect all of the sex differences in proteins contributing to pathways of interest which may create subtle cellular environmental changes, which may alter our susceptibility to a pathology across our lifetimes. However, this work has uncovered novel trends in sex-hormone interactions, such as those implied in our PPI and functional enrichment analyses, which can be used to inform the development of hypotheses for future studies [50]. We also do not have data regarding the phase of the oestrous cycle our control females were in at the time of sacrifice or know if the phase of the oestrous cycle impacts the action of Tibolone.

## 4. Materials and Methods

### 4.1. Model Preparation

Twenty-four 2-month-old C57BL/6 mice from multiple litters sourced from the Cajal Institute (Madrid, Spain) were kept under a 12 h light:12 h dark schedule. Mouse handling was performed in line with the NIH ‘Care and Use of Laboratory Animals guidelines’, and the ‘Use of Animals in Neuroscience Research’ by the Society for Neuroscience, and following the European Union guidelines (Council Directive 86/609/EEC).

Ethical approval was provided by the Institutional Animal Use and Care Committee (Comité de Ética de Experimentación Animal del Instituto Cajal) and by the Consejería del Medio Ambiente y Territorio (Comunidad de Madrid, PROEX 134/17). For the purpose of the experiment, a high number of animals would be most statistically healthy; however, given the novelty of the investigation, we chose to reduce the number of animals whilst allowing for a greater alpha value. Bilateral gonadectomy under halothane anaesthesia was performed at 2 months, with sham surgery in controls. At 3 months, 24 h before sacrifice, animals received one subcutaneous (sc) injection with 250 µg Tibolone (the human-equivalent therapeutic dose) or vehicle (corn oil). Animal sacrifice was via cervical dislocation, brains were removed and 1g of pre-frontal cortex samples were collected before being delivered to the Centro Nacional de Biotecnología (CNB-CSIC, Madrid, Spain) for proteome analysis. This resulted in 8 test groups, with *n* = 3.

### 4.2. Proteomic Dataset Production

#### 4.2.1. Protein Processing for Proteomics

All samples were lysed with 500 µL of lysis buffer (5% SDS + 125 mM TEAB + 5 mM TCEP + 10 mM CAA + protease/phosphatase inhibitors). This mixture was incubated at 60 °C for 30 min, sonicated, and then, centrifuged at 16,000× *g* for 15 min. The resulting supernatant was collected and quantified using a PIERCE 660 nm protein concentration assay. Protein digestion using an S-Trap filter (Protifi, Huntington, NY, USA) was performed with slight modifications. Briefly, 20 µg of protein of each sample was diluted to 40 µL with 5% SDS. Samples were reduced and alkylated by adding 5 mM tris(2-carboxyethyl)phosphine and 10 mM chloroacetamide for 30 min at 60 °C. Then, 12% phosphoric acid and then seven volumes of binding buffer (90% methanol; 100 mM TEAB) were added to the sample (final phosphoric acid concentration: 1.2%). After mixing, the protein solution was loaded to the S-Trap filter in two consecutive steps, separated by 2 min of centrifugation at 3000× *g*. Then, the filter was washed 3 times with 150 μL of binding buffer. Finally, samples were digested at 37 °C with Pierce MS-grade trypsin (Thermo-Fisher Scientific, Madrid, Spain) at an enzyme:substrate ratio of 1:20 (*w*/*w*) in 20 μL of a 100 mM TEAB solution. Samples were spun in the S-Trap before digestion. Flow-through was then reloaded to the top of the S-Trap column and allowed to digest in a wet chamber at 37 °C overnight. To elute peptides, two step-wise buffers were applied ((1) 40 μL of 25 mM TEAB and (2) 40 μL of 80% acetonitrile and 0.2% formic acid in H2O), separated by 2 min of centrifugation at 3000× *g* in each case. Eluted peptides were pooled and vacuum-centrifuged to dryness. Digested samples were cleaned up/desalted using StageTips with Empore 3M C18 disks (Sigma-Aldrich, Madrid, Spain) [1]. After desalting, peptide concentration was determined via Qubit™ Fluorometric Quantitation (Thermo Fisher Scientific, Waltham, MA, USA).

#### 4.2.2. Analysis via Liquid Chromatography Coupled to TRIPLE-TOF Mass Spectrometry

A 1 µg aliquot of each digested sample was subjected to 1D-nano LC ESI-MSMS analysis using a nano-liquid chromatography system (Eksigent Technologies nanoLC Ultra 1D plus, SCIEX, Foster City, CA, USA) coupled to a high-speed Triple TOF 5600 mass spectrometer (SCIEX, Foster City, CA, USA) with a Nanospray III source. The analytical column used was a silica-based reversed-phase Acquity UPLC^®^ M-Class Peptide BEH C18 Column (75 µm × 150 mm, 1.7 µm particle size and 130 Å pore size) (Waters). The trap column was a C18 Acclaim PepMapTM 100 (Thermo Scientific) (100 µm × 2 cm, 5 µm particle diameter, 100 Å pore size), switched on-line using the analytical column. The loading pump delivered a solution of 0.1% formic acid in water at 2 µL/min. The nano-pump provided a flow rate of 250 nl/min and was operated under gradient elution conditions. Peptides were separated using a 150 min gradient ranging from 2% to 90% mobile phase B (mobile phase A: 2% acetonitrile, 0.1% formic acid; mobile phase B: 100% acetonitrile, 0.1% formic acid). The injection volume was 5 µL.

Data acquisition was performed using a Triple-TOF 5600 System (SCIEX, Foster City, CA, USA). Data were acquired using ion spray voltage floating (ISVF) of 2300 V, curtain gas (CUR) of 35, interface heater temperature (IHT) of 150, ion source gas 1 (GS1) of 25 and declustering potential (DP) of 100 V. All data were acquired in information-dependent acquisition (IDA) mode using Analyst TF 1.7 software (SCIEX, Framingham, MA, USA). For IDA parameters, 0.25 s MS survey scans in the mass range of 350–1250 Da were followed by 35 MS/MS scans of 100 ms in the mass range of 100–1800 (total cycle time: 4 s). Switching criteria were set to ions greater than mass-to-charge ratio (*m*/*z*) of 350 and smaller than *m*/*z* 1250 with a charge state of 2–5 and an abundance threshold of more than 90 counts (cps). Former target ions were excluded for 15 s. IDA rolling collision energy (CE) parameter script was used for automatically controlling the CE.

Mass spectrometry data obtained were analysed using MaxQuant [51] software (v1.10.43) against the *Mus musculus* Uniprot database. Data quality was set by filtering with FDRs (false-positive rate) for both identification (FDR < 0.01 at the peptide level) and quantification (*q* value ≤ 0.05).

#### 4.2.3. Data Preparation

Raw data were processed using Perseus [52] and filtered to proteins with a unique peptide, with 2 quantified values per test group (values could be shared between overlapping groups). Values were then transformed, and the remaining values were imputed following quality check of the normal distribution of each sample.

### 4.3. Bioinformatic Analysis

#### 4.3.1. Functional Enrichment Mapping

Significantly expressed protein lists in each group were imported into cystoscope [53] (version 3.9.0) STRING protein [54] queries, within *Mus musculus,* with a confidence cut off of 0.40 for protein network formation. This was followed by functional enrichment analysis including GO biological process, GO cellular components, GO molecular functions, KEGG pathways and reactome pathways. Redundant terms were removed with a 0.5 cutoff. Related pathways were mapped using EnrichmentMap [55,56] with a node cutoff *q* value < 0.05 and an edge cutoff <0.25. Enriched functions were then clustered into curated groups [57].

#### 4.3.2. Protein–Protein Interactions (PPI)

Utilizing Metascape [21], we performed PPI enrichment analysis of our significant proteins lists. *Mus musculus* was the target species and PPI was analysed utilizing the following databases: STRING, BioGrid, OmniPath and InWeb_IM. STRING (physical score > 0.132) and BioGrid are used to identify physical interactions. The PPI networks generated include proteins with at least 1 interaction. A molecular complex detection (MCODE) algorithm then groups associated interactors that share functionality.

#### 4.3.3. Pathway and Process Enrichment Analysis

We analysed the shared functionally enriched pathways between each protein list sourced from GO biological processes, the KEGG pathway, reactome gene sets, CORUM, TRRUST, PaGenBase, WikiPathways and the PANTHER Pathway. Terms with a *p* value < 0.01, a minimum count of 3, and an enrichment factor >1.5 were then grouped into clusters. *p* values were calculated based on a hypergeometric distribution, and *q* values were calculated using the Benjamini–Hochberg procedure. Kappa scores were used as the similarity metric when performing hierarchical clustering on the enriched terms, and sub-trees with a similarity of >0.3 were considered a cluster. The most statistically significant term within a cluster was chosen to represent the cluster. A term may be found enriched in several individual protein lists, and the best *p* value among them was chosen as the final *p* value.

The clusters that were found to be of interest were used to prioritize the proteins that fell into those clusters. A subset of enriched terms was then rendered as a network plot, where terms with a similarity >0.3 were connected. We selected terms with the best *p* values from each of the 20 clusters, with the constraint that there were no more than 15 terms per cluster and no more than 250 terms in total. The network was visualized using Cytoscape, where each node represents an enriched term and is coloured first by its cluster-ID. This was performed within Metascape [21].

### 4.4. Graphing

Volcano plots were produced in R using the ggplot package [58]. Heat maps were generated using the online Heatmapper tool [59]. All PPIs, pathways and enrichment images were produced utilizing Cytoscape version 3.9.0 [53,54]. The shared pathways heat map in Figure 6, as well as the Circos plot, were generated in Metascape [21]. Bar charts were generated using GraphPad Prism version 9.3.1 for Windows, GraphPad Software, San Diego, CA, USA, www.graphpad.com. Venn diagrams were generated using an online webtool developed by the Van de Peer lab (http://bioinformatics.psb.ugent.be/webtools/Venn/, accessed on 12 May 2022).

### 4.5. Statistical Analysis

Our aim in the proteomic profile of this model was to identify subtle but clinically relevant differences between male and female mice which may contribute to sex differences in incidence across a lifetime. Sex differences are generally subtle and require a large number of test subjects to uncover [50]. This raises ethical issues of best practice—particularly around the ‘reduce’ component. As such, with our 8 groups with *n* = 3, we elected for *p* < 0.15 to identify proteins with sexually dimorphic expression which are likely to be impacted by factors of sex differences; once collected, we utilized a cutoff of *p* < 0.05 for protein–protein interactions (PPI) and *q* < 0.05 when identifying functionally enriched pathways impacted by sex-associated factors.

## 5. Conclusions

We identified that the expression of NDUFA2, NDUFA7 and UQCR10, which each appear to be vital for the efficiency of respirasome activity, has sex-specific reprogrammed responses to Tibolone dependent on gonadal hormone status. Together, this suggests that future studies on hormone replacement therapies must consider sex- and gonad-specific interactions on protein expression, particularly for studies related to mitochondrial metabolism. More studies are warranted to uncover a potential sex-specific mechanism of action behind the reprogramming of mitochondrial metabolism-associated protein expression due to hormonal deprivation, akin to menopause, via Tibolone drug repurposing. This study also supports further investigation into hormone-based precision medicine intervention for women against sex-discriminating diseases such as Alzheimer’s disease, as it is possible that protective action may be time-dependent. Given the significant risk of menopause contributing to increased incidence of many diseases in women, a better-timed hormonal intervention for the individual could, in part, ameliorate this difference.

## Data Availability

The datasets generated and/or analysed are available as Supplementary Materials in the current study.

## Supplementary Materials

The following supporting information can be downloaded at: https://www.mdpi.com/article/10.3390/ijms232314754/s1.
