# Supplementary material for: Respirasome Proteins Are Regulated by Sex-Hormone Interactions in the Brain
Source: Int J Mol Sci. 2022 Nov 25;23(23):14754. doi: 10.3390/ijms232314754 (PMC9741126; doi:10.3390/ijms232314754)
Supplement: Supplementary file 1 [file ijms-23-14754-s001.zip › Supplementary Tables.pdf]

Supplementary Table S1. The top 3 PPI-enriched function in males vs females.

PPI Cluster	Gene Ontology |Description| Log10(p)
Fully connected Cluster	GO:0051640|organelle localization|-10.3;GO:0051656|establishment of organelle localization|-8.9;GO:0097435|supramolecular fiber organization|-8.7
Translation	R-MMU-8953854|Metabolism of RNA|-9.6;R-MMU-195253|Degradation of beta-catenin by the destruction complex|-8.3;R-MMU-1799339|SRP-dependent cotranslational protein targeting to membrane|-8.0
Neuron Adhesion	R-MMU-422475|Axon guidance|-9.9;R-MMU-9675108|Nervous system development|-9.8;R-MMU-437239|Recycling pathway of L1|-8.2
Golgi-ER Transport	R-MMU-6807878|COPI-mediated anterograde transport|-5.8;R-MMU-8856688|Golgi-to-ER retrograde transport|-5.4;R-MMU-199977|ER to Golgi Anterograde Transport|-5.2
Mitochondrial Metabolism	ko00190|Oxidative phosphorylation|-11.0;mmu05012|Parkinson's disease|-10.8;ko04932|Non-alcoholic fatty liver disease (NAFLD)|-10.7
Vesicle Trafficking	R-MMU-8876198|RAB GEFs exchange GTP for GDP on RABs|-7.1;R-MMU-9007101|Rab regulation of trafficking|-6.8;R-MMU-199991|Membrane Trafficking|-4.8
Platelet Function	R-MMU-114608|Platelet degranulation|-6.8;R-MMU-76005|Response to elevated platelet cytosolic Ca2+|-6.7;R-MMU-76002|Platelet activation, signaling and aggregation|-5.8

Full list of all enriched functions is in Supplementary File S2.

Supplementary Table S2. Top 20 STRING Enriched Functions regulated by Sex.

# background genes	# genes	category	description	FDR value	p-value	term name
10283	91	GO Cellular Component	Cytoplasm	2.58E-18	1.05E-21	GO:0005737
2050	41	GO Cellular Component	Cell junction	1.97E-14	3.21E-17	GO:0030054
1583	35	GO Cellular Component	Neuron projection	2.97E-13	8.46E-16	GO:0043005
212	15	GO Cellular Component	Myelin sheath	2.81E-11	9.15E-14	GO:0043209
836	23	GO Cellular Component	Cell body	6.2E-10	3.03E-12	GO:0044297
1411	30	GO Molecular Function	Protein-containing complex binding	2.52E-09	4.81E-13	GO:0044877
1064	26	GO Biological Process	Cytoskeleton organization	1.06E-08	1.21E-12	GO:0007010
761	20	GO Cellular Component	Postsynapse	3.13E-08	2.16E-10	GO:0098794
2438	37	GO Biological Process	Regulation of cellular component organization	4.01E-08	9.15E-12	GO:0051128
4035	44	GO Cellular Component	Intracellular non-membrane-bounded organelle	2.72E-07	2.21E-09	GO:0043232
3573	40	GO Cellular Component	Cytosol	9.64E-07	9.02E-09	GO:0005829
1518	27	GO Biological Process	Establishment of localization in cell	1.73E-06	4.95E-10	GO:0051649
2806	36	GO Biological Process	Regulation of localization	5.65E-06	2.09E-09	GO:0032879
861	18	GO Cellular Component	Supramolecular fiber	6.26E-06	6.11E-08	GO:0099512
775	17	GO Cellular Component	Perinuclear region of cytoplasm	7.05E-06	7.64E-08	GO:0048471
354	12	GO Cellular Component	Cell projection membrane	7.74E-06	8.82E-08	GO:0031253
438	13	GO Cellular Component	Cell leading edge	9.32E-06	1.1E-07	GO:0031252
2021	29	GO Molecular Function	Nucleotide binding	1.29E-05	1.23E-08	GO:0000166
607	16	GO Biological Process	Regulation of neuron projection development	1.78E-05	1.65E-08	GO:0010975
521	15	GO Biological Process	Modulation of chemical synaptic transmission	1.78E-05	1.57E-08	GO:0050804

Full list of all enriched functions is in Supplementary File S3.

Supplementary Table S3. The top 3 PPI-enriched function in gonads vs gonadectomy.

PPI Cluster	Gene Ontology |Description| Log10(p);
Fully connected Cluster	GO:0032271|regulation of protein polymerization|-10.3; GO:0043254|regulation of protein-containing complex assembly|-10.2; ko01200|Carbon metabolism|-9.8
Protein Folding	GO:0006457|protein folding|-12.5;R-MMU-3371556|Cellular response to heat stress|-10.4;GO:0042026|protein refolding|-9.0
Mitochondrial Metabolism	ko00640|Propanoate metabolism|-8.5;mmu00640|Propanoate metabolism|-8.4;ko00280|Valine, leucine and isoleucine degradation|-7.5
Cytoskeleton	R-MMU-5689603|UCH proteinases|-6.9;R-MMU-5663213|RHO GTPases Activate WASPs and WAVEs|-6.1;WP523|Regulation of actin cytoskeleton|-6.0
Endocytosis	GO:0006898|receptor-mediated endocytosis|-14.2;GO:0072583|clathrin-dependent endocytosis|-14.2;R-MMU-8856828|Clathrin-mediated endocytosis|-13.8
Translation	ko03010|Ribosome|-13.0;mmu03010|Ribosome|-13.0;R-MMU-1799339|SRP-dependent cotranslational protein targeting to membrane|-12.4
Vesicle Trafficking	R-MMU-199991|Membrane Trafficking|-4.8;R-MMU-5653656|Vesicle-mediated transport|-4.7

Full list of all enriched functions is in Supplementary File S2.

Supplementary Table S4. Top 20 STRING Enriched Functions regulated by Gonadectomy.

# background genes	# genes	category	description	FDR value	p-value	term name
212	24	GO Cellular Component	Myelin sheath	6.03E-23	2.45E-26	GO:0043209
1492	36	GO Cellular Component	Synapse	1.96E-18	7.97E-22	GO:0045202
10283	89	GO Cellular Component	Cytoplasm	4.98E-18	4.05E-21	GO:0005737
1776	33	GO Cellular Component	Mitochondrion	1.44E-10	4.70E-13	GO:0005739
1779	33	Reactome Pathways	Metabolism	8.55E-09	4.92E-13	MMU-1430728
2021	33	GO Molecular Function	Nucleotide binding	2.61E-08	1.52E-11	GO:0000166
11084	66	GO Cellular Component	Intracellular organelle	3.12E-08	7.62E-11	GO:0043229
485	15	GO Cellular Component	Cytoplasmic vesicle membrane	4.98E-08	1.62E-10	GO:0030659
446	14	GO Cellular Component	Neuron to neuron synapse	1.11E-07	5.89E-10	GO:0098984
1080	20	GO Cellular Component	Somatodendritic compartment	1.16E-07	7.07E-10	GO:0036477
32	7	KEGG Pathways	Citrate cycle (TCA cycle)	1.48E-07	3.41E-10	mmu00020
689	20	GO Biological Process	Cellular amide metabolic process	1.55E-07	2.65E-11	GO:0043603
947	21	GO Molecular Function	Cytoskeletal protein binding	7.25E-07	9.71E-10	GO:0008092
223	12	GO Biological Process	Regulation of protein polymerization	9.64E-07	4.96E-10	GO:0032271
2277	32	GO Molecular Function	Enzyme binding	9.91E-07	1.52E-09	GO:0019899
244	10	GO Cellular Component	Synaptic vesicle	1.85E-06	1.86E-08	GO:0008021
19	5	GO Cellular Component	Clathrin coat of coated pit	1.85E-06	1.81E-08	GO:0030132
978	20	GO Biological Process	Regulation of protein localization	2.25E-06	1.29E-10	GO:0032880
1411	24	GO Molecular Function	Protein-containing complex binding	3.96E-06	8.74E-09	GO:0044877
1130	20	GO Biological Process	Vesicle-mediated transport	5.41E-06	1.53E-09	GO:0016192

Full list of all enriched functions is in Supplementary File S3.

Supplementary Table S5. The top 3 PPI-enriched function in Tibolone vs Control.

PPI Cluster	Gene Ontology |Description| Log10(p);
Fully connected Cluster	GO:0006091|generation of precursor metabolites and energy|-11.9;ko05010|Alzheimer's disease|-11.3;mmu05010|Alzheimer's disease|-11.3
Transcription Regulation	R-MMU-382551|Transport of small molecules|-6.8;R-MMU-195253|Degradation of beta-catenin by the destruction complex|-6.6;R-MMU-8878159|Transcriptional regulation by RUNX3|-6.6
Citric Acid Cycle	R-MMU-1428517|The citric acid (TCA) cycle and respiratory electron transport|-9.3;GO:0009060|aerobic respiration|-7.5;GO:0006091|generation of precursor metabolites and energy|-6.9
Translation	R-MMU-1799339|SRP-dependent cotranslational protein targeting to membrane|-22.1;R-MMU-975956|Nonsense Mediated Decay (NMD) independent of the Exon Junction Complex (EJC)|-22.0;R-MMU-72689|Formation of a pool of free 40S subunits|-21.6
Mitochondrial Metabolism	GO:0015980|energy derivation by oxidation of organic compounds|-6.3;R-MMU-109606|Intrinsic Pathway for Apoptosis|-5.9;GO:0009060|aerobic respiration|-5.8
mRNA Splicing	R-MMU-72163|mRNA Splicing - Major Pathway|-13.7;R-MMU-72172|mRNA Splicing|-13.6;R-MMU-72203|Processing of Capped Intron-Containing Pre-mRNA|-12.8
Endocytosis	R-MMU-8856828|Clathrin-mediated endocytosis|-9.6;GO:0072583|clathrin-dependent endocytosis|-9.2;R-MMU-199991|Membrane Trafficking|-8.7
Statin Pathways	WP1|Statin pathway|-9.2;GO:0010875|positive regulation of cholesterol efflux|-8.5;R-MMU-2173782|Binding and Uptake of Ligands by Scavenger Receptors|-8.4

Full list of all enriched functions is in Supplementary File S2.

Supplementary Table S6. Top 20 STRING Enriched Functions regulated by Tibolone.

# background genes	# genes	category	description	FDR value	p-value	term name
10283	96	GO Cellular Component	Cytoplasm	3.11E-18	1.27E-21	GO:0005737
1492	30	GO Cellular Component	Synapse	2.76E-16	1.63E-19	GO:0045202
212	18	GO Cellular Component	Myelin sheath	4.03E-14	4.32E-17	GO:0043209
1776	37	GO Cellular Component	Mitochondrion	3.11E-12	7.60E-15	GO:0005739
761	24	GO Cellular Component	Postsynapse	7.38E-11	2.70E-13	GO:0098794
3573	49	GO Cellular Component	Cytosol	1.37E-10	5.58E-13	GO:0005829
122	12	KEGG Pathways	Carbon metabolism	8.96E-10	1.97E-12	mmu01200
4895	56	GO Cellular Component	Protein-containing complex	1.47E-09	7.17E-12	GO:0032991
32	8	KEGG Pathways	Citrate cycle (TCA cycle)	3.23E-09	1.42E-11	mmu00020
167	14	Reactome Pathways	The citric acid (TCA) cycle and respiratory electron transport	3.39E-09	1.95E-13	MMU-1428517
697	21	GO Cellular Component	Mitochondrial envelope	4.17E-09	2.38E-11	GO:0005740
1083	25	GO Cellular Component	Membrane protein complex	7.74E-09	5.98E-11	GO:0098796
1536	29	KEGG Pathways	Metabolic pathways	2.14E-08	1.41E-10	mmu01100
501	14	GO Cellular Component	Glutamatergic synapse	2.14E-08	7.56E-11	GO:0098978
319	16	GO Biological Process	Generation of precursor metabolites and energy	4.93E-08	5.64E-12	GO:0006091
2472	36	GO Cellular Component	Cell projection	6.43E-08	5.76E-10	GO:0042995
449	16	GO Cellular Component	Postsynaptic specialization	7.66E-08	7.17E-10	GO:0099572
264	13	KEGG Pathways	Prion disease	8.83E-08	7.78E-10	mmu05020
1080	23	GO Cellular Component	Somatodendritic compartment	1.82E-07	1.85E-09	GO:0036477
636	14	GO Cellular Component	Presynapse	2.94E-07	1.56E-09	GO:0098793

Full list of all enriched functions is in Supplementary File S3.

Supplementary Table S7. The top 3 PPI-enriched function regulated by Sex-Gonad interactions.

PPI Cluster	Gene Ontology |Description| Log10(p);
Fully connected Cluster	R-MMU-927802|Nonsense-Mediated Decay (NMD)|-9.5;R-MMU-975957|Nonsense Mediated Decay (NMD) enhanced by the Exon Junction Complex (EJC)|-9.5;R-MMU-1799339|SRP-dependent cotranslational protein targeting to membrane|-9.1
Protein Folding	R-MMU-927802|Nonsense-Mediated Decay (NMD)|-21.8;R-MMU-975957|Nonsense Mediated Decay (NMD) enhanced by the Exon Junction Complex (EJC)|-21.8;R-MMU-1799339|SRP-dependent cotranslational protein targeting to membrane|-19.8
Mitochondrial Metabolism	R-MMU-611105|Respiratory electron transport|-16.5; WP295|Electron transport chain|-16.3; CORUM:381|Respiratory chain complex I, mitochondrial|-15.8
Cytoskeleton	WP163|Cytoplasmic ribosomal proteins|-13.2; ko03010|Ribosome| -11.6; mmu03010|Ribosome|-11.6
Endocytosis	R-MMU-195253|Degradation of beta-catenin by the destruction complex|-12.2; R-MMU-201681|TCF dependent signaling in response to WNT|-10.7; R-MMU-195721|Signaling by WNT|-9.8
Translation	mmu03050|Proteasome|-8.0;ko03050|Proteasome|-8.0;R-MMU-1236978|Cross-presentation of soluble exogenous antigens (endosomes)|-7.9
Protein Trafficking	R-MMU-72163|mRNA Splicing - Major Pathway|-6.2;R-MMU-72172|mRNA Splicing|-6.2;R-MMU-72203|Processing of Capped Intron-Containing Pre-mRNA|-5.9
Protein Phosphorylation	R-MMU-8957275|Post-translational protein phosphorylation|-6.8;R-MMU-381426|Regulation of Insulin-like Growth Factor (IGF) transport and uptake by Insulin-like Growth Factor Binding Proteins (IGFBPs)|-6.7

Full list of all enriched functions is in Supplementary File S2.

Supplementary Table S8. Top 20 STRING Enriched Functions regulated by Sex and Gonadectomy.

# background genes	# genes	category	description	FDR value	p-value	term name
10283	104	GO Cellular Component	Cytoplasm	4.14E-17	1.68E-20	GO:0005737
1492	43	GO Cellular Component	Synapse	4.14E-17	2.17E-20	GO:0045202
2387	43	GO Cellular Component	Plasma membrane bounded cell projection	7.30E-11	3.86E-13	GO:0120025
3573	50	GO Cellular Component	Cytosol	3.55E-09	2.60E-11	GO:0005829
636	20	GO Cellular Component	Presynapse	4.09E-08	3.16E-10	GO:0098793
2973	42	GO Cellular Component	Organelle membrane	1.96E-07	1.76E-09	GO:0031090
4895	56	GO Cellular Component	Protein-containing complex	2.33E-07	2.18E-09	GO:0032991
1962	35	GO Molecular Function	Identical protein binding	2.92E-07	1.68E-10	GO:0042802
1776	31	GO Cellular Component	Mitochondrion	4.30E-07	4.20E-09	GO:0005739
743	20	GO Cellular Component	Neuronal cell body	4.30E-07	4.24E-09	GO:0043025
99	9	GO Cellular Component	Cytosolic ribosome	6.22E-07	6.83E-09	GO:0022626
2406	38	GO Molecular Function	Small molecule binding	8.88E-07	6.80E-10	GO:0036094
4054	48	GO Cellular Component	Non-membrane-bounded organelle	2.05E-06	2.34E-08	GO:0043228
212	11	GO Cellular Component	Myelin sheath	2.88E-06	3.40E-08	GO:0043209
58	7	GO Cellular Component	Intercalated disc	4.74E-06	5.79E-08	GO:0014704
1310	24	GO Cellular Component	Plasma membrane region	1.04E-05	1.43E-07	GO:0098590
1131	22	GO Cellular Component	Organelle envelope	1.34E-05	1.91E-07	GO:0031967
1779	31	Reactome Pathways	Metabolism	1.66E-05	4.37E-09	MMU-1430728
399	13	GO Cellular Component	Distal axon	2.22E-05	3.44E-07	GO:0150034
58	7	KEGG Pathways	Endocrine and other factor-regulated calcium reabsorption	2.63E-05	5.79E-08	mmu04961

Full list of all enriched functions is in Supplementary File S3.

Supplementary Table S9. The top 3 PPI-enriched function regulated by Sex-Tibolone interactions.

PPI Cluster	Gene Ontology |Description| Log10(p);
Fully connected Cluster	mmu05012|Parkinson's disease|-9.4;R-MMU-1428517|The citric acid (TCA) cycle and respiratory electron transport|-8.6;WP295|Electron transport chain|-8.4
Citric Acid Cycle	M00009|Citrate cycle (TCA cycle, Krebs cycle)|-6.4;mmu_M00009|Citrate cycle (TCA cycle, Krebs cycle)|-6.4;ko00020|Citrate cycle (TCA cycle)|-6.0
Translation	WP163|Cytoplasmic ribosomal proteins|-12.6;ko03010|Ribosome|-11.0;mmu03010|Ribosome|-11.0
Electron Transport Chain	CORUM:381|Respiratory chain complex I, mitochondrial|-16.6;CORUM:382|Respiratory chain complex I, mitochondrial|-16.4;R-MMU-6799198|Complex I biogenesis|-15.5
LTD and E2 signalling	ko04730|Long-term depression|-9.0;mmu04730|Long-term depression|-9.0;ko04915|Estrogen signaling pathway|-8.2
AKT and Proteosome	R-MMU-1257604|PIP3 activates AKT signaling|-7.9;R-MMU-9006925|Intracellular signaling by second messengers|-7.7;ko03050|Proteasome|-7.4

Full list of all enriched functions is in Supplementary File S2.

Supplementary Table S10. Top 20 STRING Enriched Functions regulated by Sex and Tibolone.

# background genes	# genes	category	description	FDR value	p-value	term name
10283	120	GO Cellular Component	Cytoplasm	1.22E-23	4.95E-27	GO:0005737
1492	46	GO Cellular Component	Synapse	7.48E-18	6.09E-21	GO:0045202
1776	42	GO Cellular Component	Mitochondrion	2.16E-12	5.28E-15	GO:0005739
2472	49	GO Cellular Component	Cell projection	3.81E-12	1.09E-14	GO:0042995
359	19	KEGG Pathways	Alzheimer disease	5.17E-10	1.14E-12	mmu05010
3573	54	GO Cellular Component	Cytosol	2.33E-09	1.52E-11	GO:0005829
1779	39	Reactome Pathways	Metabolism	1.23E-08	7.10E-13	MMU-1430728
2973	47	GO Cellular Component	Organelle membrane	1.94E-08	1.42E-10	GO:0031090
4895	62	GO Cellular Component	Protein-containing complex	3.89E-08	3.48E-10	GO:0032991
1962	38	GO Molecular Function	Identical protein binding	1.01E-07	5.83E-11	GO:0042802
435	17	GO Cellular Component	Mitochondrial inner membrane	1.65E-07	1.68E-09	GO:0005743
2021	38	GO Molecular Function	Nucleotide binding	1.78E-07	1.36E-10	GO:0000166
501	18	GO Cellular Component	Glutamatergic synapse	1.82E-07	1.93E-09	GO:0098978
3822	55	GO Biological Process	Regulation of biological quality	5.23E-07	5.97E-11	GO:0065008
1080	25	GO Cellular Component	Somatodendritic compartment	6.67E-07	7.33E-09	GO:0036477
2115	39	GO Biological Process	Cellular localization	7.37E-07	1.26E-10	GO:0051641
212	12	GO Cellular Component	Myelin sheath	8.64E-07	1.01E-08	GO:0043209
1872	33	GO Cellular Component	Cytoplasmic vesicle	1.23E-06	1.51E-08	GO:0031410
104	9	GO Cellular Component	Oxidoreductase complex	1.93E-06	2.52E-08	GO:1990204
414	15	GO Cellular Component	Postsynaptic density	2.91E-06	4.26E-08	GO:0014069

Full list of all enriched functions is in Supplementary File S3.

Supplementary Table S11. The top 3 PPI-enriched function regulated by Gonad-Tibolone interactions.

PPI Cluster	Gene Ontology |Description| Log10(p);
Fully connected Cluster	GO:0032271|regulation of protein polymerization|-7.9;GO:0032535|regulation of cellular component size|-7.3;GO:0097435|supramolecular fiber organization|-7.1
Antigen Processing	R-MMU-983168|Antigen processing: Ubiquitination & Proteasome degradation|-8.6;R-MMU-983169|Class I MHC mediated antigen processing & presentation|-8.0;R-MMU-1280218|Adaptive Immune System|-7.5
Translation	ko03010|Ribosome|-12.5;mmu03010|Ribosome|-12.5;GO:0006412|translation|-12.4
RAB Signalling	R-MMU-8873719|RAB geranylgeranylation|-15.5;R-MMU-9007101|Rab regulation of trafficking|-10.6;R-MMU-8876198|RAB GEFs exchange GTP for GDP on RABs|-8.3
Endocytosis	R-MMU-8856828|Clathrin-mediated endocytosis|-10.2;R-MMU-199991|Membrane Trafficking|-7.2;R-MMU-5653656|Vesicle-mediated transport|-7.0
Electron Transport Chain	GO:0042773|ATP synthesis coupled electron transport|-12.5;GO:0022904|respiratory electron transport chain|-11.9;GO:0022900|electron transport chain|-11.7
Cytoskeleton	GO:0045104|intermediate filament cytoskeleton organization|-7.8;GO:0045103|intermediate filament-based process|-7.8

Full list of all enriched functions is in Supplementary File S2.

Supplementary Table S12. Top 20 STRING Enriched Functions regulated by Gonadectomy and Tibolone.

# background genes	# genes	category	description	FDR value	p-value	term name
1492	63	GO Cellular Component	Synapse	1.49E-27	6.05E-31	GO:0045202
10283	145	GO Cellular Component	Cytoplasm	9.46E-27	7.70E-30	GO:0005737
1583	50	GO Cellular Component	Neuron projection	2.05E-16	8.33E-19	GO:0043005
501	30	GO Cellular Component	Glutamatergic synapse	5.89E-16	2.69E-18	GO:0098978
446	24	GO Cellular Component	Neuron to neuron synapse	1.24E-11	8.09E-14	GO:0098984
483	25	GO Molecular Function	Structural molecule activity	9.36E-11	5.37E-14	GO:0005198
212	17	GO Cellular Component	Myelin sheath	1.70E-10	1.17E-12	GO:0043209
3573	64	GO Cellular Component	Cytosol	2.45E-10	1.99E-12	GO:0005829
2438	54	GO Biological Process	Regulation of cellular component organization	6.11E-10	6.98E-14	GO:0051128
4054	68	GO Cellular Component	Non-membrane-bounded organelle	6.52E-10	5.67E-12	GO:0043228
4895	76	GO Cellular Component	Protein-containing complex	6.76E-10	6.33E-12	GO:0032991
244	17	GO Cellular Component	Synaptic vesicle	9.81E-10	9.58E-12	GO:0008021
1415	36	GO Cellular Component	Whole membrane	6.52E-09	7.69E-11	GO:0098805
1776	39	GO Cellular Component	Mitochondrion	5.45E-08	7.19E-10	GO:0005739
1310	33	GO Cellular Component	Plasma membrane region	5.45E-08	7.10E-10	GO:0098590
4769	74	GO Biological Process	Cellular component organization	6.81E-08	1.58E-11	GO:0016043
4646	73	GO Biological Process	Localization	6.81E-08	1.32E-11	GO:0051179
2358	48	GO Biological Process	Nervous system development	1.58E-07	5.42E-11	GO:0007399
753	24	GO Cellular Component	Dendrite	1.89E-07	2.70E-09	GO:0030425
4668	68	GO Cellular Component	Cell periphery	2.08E-07	3.13E-09	GO:0071944

Supplementary Table S13. The top 3 PPI-enriched function regulated by Sex-Gonad-Tibolone interactions.

PPI Cluster	Gene Ontology |Description| Log10(p);
Fully connected Cluster	ko05010|Alzheimer's disease|-9.6;mmu05010|Alzheimer's disease|-9.6;R-MMU-611105|Respiratory electron transport|-8.6
Cellular Metabolism	R-MMU-422356|Regulation of insulin secretion|-8.4;R-MMU-163685|Integration of energy metabolism|-7.9;R-MMU-418217|G beta:gamma signalling through PLC beta|-7.5
Electron Transfer Chain	R-MMU-611105|Respiratory electron transport|-16.5;WP295|Electron transport chain|-16.3;CORUM:381|Respiratory chain complex I, mitochondrial|-15.8
Translation	WP163|Cytoplasmic ribosomal proteins|-13.2;ko03010|Ribosome|-11.6;mmu03010|Ribosome|-11.6
WNT Signalling	R-MMU-195253|Degradation of beta-catenin by the destruction complex|-12.2;R-MMU-201681|TCF dependent signaling in response to WNT|-10.7;R-MMU-195721|Signaling by WNT|-9.8
Prion Disease	ko05020|Prion diseases|-7.8;mmu05020|Prion diseases|-7.6;ko04514|Cell adhesion molecules (CAMs)|-5.7

Full list of all enriched functions is in Supplementary File S2.

Supplementary Table S14. Top 20 STRING Enriched Functions regulated by Sex, Gonadectomy and Tibolone.

# background genes	# genes	category	description	FDR value	p-value	term name
1492	65	GO Cellular Component	Synapse	1.52E-34	6.18E-38	GO:0045202
10283	121	GO Cellular Component	Cytoplasm	1.78E-20	2.90E-23	GO:0005737
501	26	GO Cellular Component	Glutamatergic synapse	6.30E-14	2.82E-16	GO:0098978
1080	34	GO Cellular Component	Somatodendritic compartment	1.57E-12	7.64E-15	GO:0036477
446	23	GO Cellular Component	Neuron to neuron synapse	3.52E-12	2.00E-14	GO:0098984
4895	69	GO Cellular Component	Protein-containing complex	1.81E-10	1.47E-12	GO:0032991
2973	52	GO Cellular Component	Organelle membrane	2.04E-10	1.79E-12	GO:0031090
264	17	GO Cellular Component	Exocytic vesicle	2.40E-10	2.35E-12	GO:0070382
1310	32	GO Cellular Component	Plasma membrane region	3.22E-09	3.93E-11	GO:0098590
212	14	GO Cellular Component	Myelin sheath	1.23E-08	1.65E-10	GO:0043209
113	11	GO Cellular Component	Schaffer collateral - CA1 synapse	2.54E-08	3.62E-10	GO:0098685
3382	55	GO Biological Process	Transport	4.68E-08	5.34E-12	GO:0006810
143	12	KEGG Pathways	Retrograde endocannabinoid signaling	5.77E-08	2.73E-10	mmu04723
359	17	KEGG Pathways	Alzheimer disease	5.77E-08	2.29E-10	mmu05010
1872	36	GO Cellular Component	Cytoplasmic vesicle	8.49E-08	1.24E-09	GO:0031410
791	23	GO Cellular Component	Axon	8.85E-08	1.39E-09	GO:0030424
3573	52	GO Cellular Component	Cytosol	9.53E-08	1.60E-09	GO:0005829
102	10	GO Cellular Component	GABA-ergic synapse	1.23E-07	2.21E-09	GO:0098982
1776	34	GO Cellular Component	Mitochondrion	2.49E-07	4.56E-09	GO:0005739
522	20	GO Biological Process	Regulation of trans-synaptic signaling	6.78E-07	1.94E-10	GO:0099177

Supplementary Table S15. Top shared proteins, minimum 4/7 hits across sex, gonads, tibolone and interactions.

Gene id	Protein name	Gonad-Tibolone	Gonad	Sex-Gonad	Sex-Gonad-Tibolone	Sex-Tibolone	Sex	Tibolone
210274	Shank2	1	1	1	1	0	1	0
66588	Cmpk1	0	1	1	1	0	1	1
13383	Dlg1	1	1	0	0	1	1	1
57440	Ehd3	0	1	1	1	1	1	0
68278	Ddx39	1	1	0	0	0	1	1
13589	Mapre1	1	1	0	0	0	1	1
225887	Ndufs8	1	1	1	1	0	0	0
243616	Slc6a11	1	0	0	1	1	1	0
12408	Cbr1	1	0	0	1	1	1	0
68051	Nutf2	1	1	0	1	0	0	1
11363	Acadl	1	0	1	0	1	1	0
108069	Grm3	1	0	1	1	0	0	1
11771	Ap2a1	0	1	1	1	0	1	0
67059	Ola1	1	1	0	0	0	1	1
17967	Ncam1	0	0	0	1	1	1	1
20527	Slc2a3	0	1	0	1	1	0	1
54127	Rps28	0	0	0	1	1	1	1
13167	Dbi	0	1	0	1	1	1	0
12331	Cap1	1	1	0	0	0	1	1
11765	Ap1g1	0	1	0	0	1	1	1
74868	Tmem65	0	1	1	0	1	1	0
76308	Rab1b	1	0	0	1	1	1	0
18293	Ogdh	1	1	1	0	0	0	1

Full list of all enriched functions is in Supplementary File S4.

Supplementary Table S16. Top 20 Clusters of Enriched functions, represented by the most significantly enriched function per cluster.

Category	Term	Description	LogP	Log(q-value)	Input Proteins / Proteins in Term
KEGG Pathway	ko05010	Alzheimer's disease	-26.3099	-22.329	44/175
Reactome Gene Sets	R-MMU-8953854	Metabolism of RNA	-25.4256	-21.746	74/546
Reactome Gene Sets	R-MMU-2262752	Cellular responses to stress	-22.0701	-19.019	66/499
GO Biological Processes	GO:1903827	regulation of cellular protein localization	-21.1179	-18.137	69/564
GO Biological Processes	GO:0099003	vesicle-mediated transport in synapse	-20.9081	-17.969	46/256
GO Biological Processes	GO:0099177	regulation of trans-synaptic signaling	-20.652	-17.761	75/669
GO Biological Processes	GO:0032990	cell part morphogenesis	-20.5667	-17.716	80/753
GO Biological Processes	GO:0072657	protein localization to membrane	-19.5269	-16.764	69/602
GO Biological Processes	GO:0032535	regulation of cellular component size	-18.3142	-15.623	55/418
Reactome Gene Sets	R-MMU-5653656	Vesicle-mediated transport	-17.8161	-15.147	64/566
Reactome Gene Sets	R-MMU-112315	Transmission across Chemical Synapses	-17.4014	-14.773	36/188
KEGG Pathway	ko01200	Carbon metabolism	-17.2198	-14.628	29/118
GO Biological Processes	GO:0050808	synapse organization	-17.0503	-14.467	58/491
GO Biological Processes	GO:0098655	cation transmembrane transport	-16.7635	-14.198	75/781
Reactome Gene Sets	R-MMU-6798695	Neutrophil degranulation	-16.6403	-14.091	58/501

Reactome Gene Sets	R-MMU-9716542	Signaling by Rho GTPases, Miro GTPases and RHOBTB3	-15.4032	-12.979	66/667
Reactome Gene Sets	R-MMU-422475	Axon guidance	-15.255	-12.848	39/256
KEGG Pathway	mmu04720	Long-term potentiation	-12.5916	-10.447	19/68
GO Biological Processes	GO:0051640	organelle localization	-12.2354	-10.144	54/559
Reactome Gene Sets	R-MMU-8856828	Clathrin-mediated endocytosis	-11.9987	-9.924	25/134

Full list of all enriched functions is in Supplementary File S4.

Supplementary Table S17. Alzheimer's cluster of enriched functions.

Category	Term	Description	LogP	Log(q-value)	Input Proteins / Proteins in Term
KEGG Pathway	ko05010	Alzheimer's disease	-26.3099	-22.329	44/175
KEGG Pathway	mmu05010	Alzheimer's disease	-26.3099	-22.329	44/175
Reactome Gene Sets	R-MMU-1428517	The citric acid (TCA) cycle and respiratory electron transport	-25.9495	-22.145	43/169
KEGG Pathway	ko05016	Huntington's disease	-22.5717	-19.440	42/192
KEGG Pathway	mmu05016	Huntington's disease	-22.5717	-19.440	42/192
GO Biological Processes	GO:0009060	aerobic respiration	-20.7288	-17.809	37/161
GO Biological Processes	GO:0006091	generation of precursor metabolites and energy	-20.6453	-17.761	59/428
KEGG Pathway	ko00190	Oxidative phosphorylation	-20.5743	-17.716	34/134
GO Biological Processes	GO:0045333	cellular respiration	-19.6785	-16.888	41/215
KEGG Pathway	mmu05012	Parkinson's disease	-18.5047	-15.782	33/144
GO Biological Processes	GO:0015980	energy derivation by oxidation of organic compounds	-18.496	-15.782	47/305
WikiPathways	WP295	Electron transport chain	-17.3357	-14.717	27/99
Reactome Gene Sets	R-MMU-163200	Respiratory electron transport, ATP synthesis by chemiosmotic coupling, and heat production by uncoupling proteins.	-17.2198	-14.628	29/118

Reactome Gene Sets	R-MMU-611105	Respiratory electron transport	-16.8831	-14.309	26/94
KEGG Pathway	mmu00190	Oxidative phosphorylation	-15.5254	-13.089	34/190
GO Biological Processes	GO:0046034	ATP metabolic process	-14.3315	-11.984	38/259
GO Biological Processes	GO:0006119	oxidative phosphorylation	-13.7829	-11.488	25/113
CORUM	CORUM:382	Respiratory chain complex I, mitochondrial	-13.4784	-11.205	16/40
CORUM	CORUM:381	Respiratory chain complex I, mitochondrial	-12.7759	-10.594	15/37
GO Biological Processes	GO:0022900	electron transport chain	-12.6379	-10.490	22/95
GO Biological Processes	GO:0022904	respiratory electron transport chain	-12.4724	-10.355	21/87
KEGG Pathway	ko04932	Non-alcoholic fatty liver disease (NAFLD)	-10.8009	-8.852	25/151
GO Biological Processes	GO:0042773	ATP synthesis coupled electron transport	-10.5914	-8.668	17/67
WikiPathways	WP1248	Oxidative phosphorylation	-10.4833	-8.569	16/59
KEGG Pathway	mmu04932	Non-alcoholic fatty liver disease (NAFLD)	-10.2893	-8.388	26/172
GO Biological Processes	GO:0042775	mitochondrial ATP synthesis coupled electron transport	-9.78985	-7.939	16/65
GO Biological Processes	GO:0019646	aerobic electron transport chain	-9.08302	-7.290	14/53
GO Biological Processes	GO:0010257	NADH dehydrogenase complex assembly	-8.62936	-6.871	14/57
GO Biological Processes	GO:0032981	mitochondrial respiratory chain complex I assembly	-8.62936	-6.871	14/57
Reactome Gene Sets	R-MMU-6799198	Complex I biogenesis	-8.62936	-6.871	14/57

GO Biological Processes	GO:0006120	mitochondrial electron transport, NADH to ubiquinone	-6.70989	-5.078	9/29
GO Biological Processes	GO:0033108	mitochondrial respiratory chain complex assembly	-6.6424	-5.021	15/92

Full list of all enriched functions is in Supplementary File S4.

Supplementary Table S18. The top 3 PPI-enriched function regulated by merged lists of proteins regulated by Sex, Gonads, Tibolone and interactions.

PPI Cluster	Gene Ontology |Description| Log10(p);
Fully connected Cluster	GO:0009060|aerobic respiration|-13.3;mmu05010|Alzheimer's disease|-12.6;ko05010|Alzheimer's disease|-12.6
Oxidative Phosphorylation	ko00190|Oxidative phosphorylation|-16.7;mmu00190|Oxidative phosphorylation|-14.7;mmu05016|Huntington's disease|-13.1
RAF/MAP Kinase Cascade	R-MMU-5673000|RAF activation|-7.7;GO:0051668|localization within membrane|-7.4;R-MMU-5673001|RAF/MAP kinase cascade|-7.2
Translation	R-MMU-1799339|SRP-dependent cotranslational protein targeting to membrane|-22.7;R-MMU-975956|Nonsense Mediated Decay (NMD) independent of the Exon Junction Complex (EJC)|-22.6;WP163|Cytoplasmic ribosomal proteins|-22.3
Muscle Contraction	GO:0003012|muscle system process|-5.6;GO:0006936|muscle contraction|-4.6;ko04921|Oxytocin signaling pathway|-4.1
Citric Acid Cycle	GO:0009060|aerobic respiration|-6.6;R-MMU-1428517|The citric acid (TCA) cycle and respiratory electron transport|-6.5;GO:0045333|cellular respiration|-6.1
Dicarboxylic Acid Metabolic Process	GO:0043648|dicarboxylic acid metabolic process|-7.1
Cytoskeleton	GO:0030048|actin filament-based movement|-6.6;GO:0007015|actin filament organization|-5.0;GO:0030036|actin cytoskeleton organization|-4.4

Full list of all enriched functions is in Supplementary File S2.
